# Inpatient hospital fatality related to coding (ICD-9-CM) of the influenza diagnosis in Spain (2009–2015)

**DOI:** 10.1186/s12879-019-4308-5

**Published:** 2019-08-07

**Authors:** J. M. San-Román-Montero, R. Gil Prieto, C. Gallardo Pino, J. Hinojosa Mena, A. Zapatero Gaviria, A. Gil de Miguel

**Affiliations:** 1 Department of Medicine and Surgery, Psychology, Preventive Medicine and Public Health and Immunology, Medical Microbiology and Nursing and Stomatology, Universidad Rey Juan Carlos, Avenida de Atenas s/n. Alcorcón, 28922 Madrid, Spain; 2 Servicio de Medicina Interna, Hospital Universitario de Fuenlabrada, Fuenlabrada, Madrid, Spain

**Keywords:** Influenza virus, Epidemiology, Hospitalizations, Inhospital mortality

## Abstract

**Background:**

To analyze hospitalization episodes with an ICD-9 diagnosis code of influenza (codes 487 and 488) in any diagnostic position from 2009 to 2015 in the Spanish hospital surveillance system.

**Methods:**

Information about age, length of stay in hospital, mortality, comorbidity with an influenza diagnosis code between 1 October 2009 and 30 September 2015 was obtained from the National Surveillance System for Hospital Data (Conjunto Mínimo Básico de Datos, CMBD).

**Results:**

52,884 hospital admissions were obtained. A total of 24,527 admissions corresponded to diagnoses ICD-9 code 487 (46.4%), and 28,357 (53.6%) corresponded to ICD-9 code 488. The global hospitalization rates were 8.7 and 10.6 per 100,000 people, respectively. Differences between the two diagnostic groups were found for each of the six analyzed seasons. The diagnostic ICD-9-CM 488, male gender, and high-risk patients classified by risk vaccination groups showed direct relationship with inpatient hospital death.

**Conclusions:**

Influenza diagnosis was present in a significant number of hospital admissions. The code used for diagnosis (ICD-9-CM 488), male sex, age groups and associated risk clinical conditions showed a direct relationship with inpatient hospital fatality.

## Background

Influenza is an infectious disease that mainly occurs with respiratory symptoms. However, a host of variables broadens the clinical spectrum, which ranges from mild forms to other, more serious hospital admission subsidiaries that can cause death of the patient.

In 2009, the World Health Organization warned of a new influenza pandemic caused by a new type A subtype H1N1 influenza virus (A(H1N1)pdm09) [1]. Currently, the virus coexists with other A and B serotypes in seasonal epidemics, and influenza is still responsible for high morbidity and mortality. In the United States, influenza is estimated to have been responsible for between 9.2 and 35.6 million cases of illness since 2010, which have resulted in approximately 140,000 to 710,000 hospitalizations and 12,000 to 56,000 deaths [2]. Data from the European Centre for Disease Prevention and Control suggest that approximately 40,000 people die prematurely each year in Europe from causes directly related to influenza infection [3]. In Spain, the cumulative incidence of influenza adjusted for age has varied in recent years between 2781 cases per 100,000 people in the 2009–2010 season and 1649 cases per 100,000 people in the 2016–2017 season [4]. In this latter season, patients over 65 years of age accounted for almost 75% of severe hospitalized cases of confirmed influenza. The inpatient fatality rate was 15% of the confirmed influenza cases, and 85% of the deaths occurred in the age group older than 65 years [4].

In this study, we aimed to assess differences of the burden of severe or complicated influenza illness and inpatient hospital fatality in each influenza season from 2009 to 2015 through the hospital discharge database. To this end, the two codes in use since 2009 for the diagnosis of influenza were considered: infections caused by influenza (ICD-9-CM 487) and those caused by certain influenza viruses (ICD-9-CM 488), mainly 2009 influenza H1N1 (ICD-9-CM 488.1). Finally, differences between the groups were evaluated.

## Methods

This analytical study assessed the database of the national hospital data system (Minimum Basic Data Set; Conjunto Mínimo Básico de Datos, CMBD), which is developed annually by the Ministry of Health, Consumption and Social Welfare of Spain. The CMBD includes information on hospital discharges using a list of clinical codes to establish the diagnosis that justified the admission based on the Spanish version of the International Classification of Diseases, 9th Revision, Clinical Modification (ICD-9-CM). The CMBD covers approximately 98% of public hospitals, and approximately 99.5% of the Spanish population is covered by healthcare. When necessary, the population figures obtained from the projection of the Spanish census from 2009 to 2015 provided by the National Statistical Centre were used as a denominator, and we assumed that the age distribution of the population with hospital coverage was equal to the general population.

All hospital discharges over a 6-season period (from 1 October 2009 to 30 September 2015) were recorded if they included a diagnosis of influenza in any diagnostic position. The episodes were subdivided into two groups according to the diagnostic code used. The first group (group 487) corresponded to episodes of influenza (ICD-9-CM 487), and the second group (group 488) included those caused by certain influenza viruses (ICD-9-CM 488). For each record, data were collected for variables including the sex, length of hospital stay, diagnosis, and outcome. ICD-9-CM 487 mainly includes seasonal influenza, influenza A(H3N2), Influenza A or influenza not otherwise specified or not coded under another concept and constitutes a significant number of admissions in which the diagnosis of influenza may or may not have been confirmed by a laboratory test. This code also includes cases in which the doctor has decided to include the diagnosis in the discharge report, because the patient reports an episode compatible with a clinical complication of influenza infection. Conversely, ICD-9-CM 488 is defined as the diagnosis of influenza due to certain identified influenza viruses, such as the avian influenza virus A/H5N1, other influenza due to new influenza A viruses and especially the diagnosis of influenza due to influenza A virus subtype H1N1 (A (H1N1) pdm09) (ICD-9-CM 488.1). Unlike code 487, the diagnosis must be recorded as such in the clinical history for the episode to be coded as 488.1, which requires laboratory confirmation [5]. Due the absence of reports of influenza virus A/H5N1 in Spain during the study we assume ICD-9-CM 488 code as influenza A virus subtype H1N1 (A (H1N1) pdm09) [5]. Regardless, since H1N1pdm09 was considered a seasonal strain after 2010, we can’t exclude that in code 487 we also find H1N1pdm09 cases.

The average number of hospitalizations per year and their age distributions, the annual incidence of hospital admissions (number of admissions per 100,000 people), the average length of stay in hospital days, the mortality rate (number of deaths per 100,000 people) and the Inpatient hospital fatality (number of deaths per inpatient population, %) were calculated. In addition, other clinical conditions described in the discharge report were analyzed and classified using the ICD-9 codes as suggested by European Centre for Disease Prevention and Control [6]. At least, for a multivariate analysis, patients were categorized in separate groups according to age and underlying conditions criteria, which are those included in the indication for influenza vaccination resulting four groups; a) patients between 2 years and 65 years without underlying clinical conditions, b) patients between 2 years and 65 years with clinical underlying condition, c) patients less than 2 years and d) patients over 65 years.

In all statistical tests, the level of significance used was *p* < 0.05. The statistical analyses were performed using the statistical package SPSS for Windows, version 22.0 (Chicago, IL, USA).

## Results

During the six seasons analyzed (2009–2010 to 2014–2015), 52,884 hospital discharges that included influenza code in any diagnostic position were recorded. A total of 24,527 entries (46.4%) were codified for ICD-9-CM 487 and 28,357 episodes (53.6%) for ICD-9-CM 488.

A total of 27,596 admissions (46.4%) were male patients, of whom 46% were in group 487, and 54% were in group 488. For females, 46.8% were in group 487, and 53.2% were in group 488. No significant differences were found in sex and its distribution by diagnostic group (*p* = 0.63). The global median age was 52.7 years (IR 49.6 years) without significant differences between males and females. However, in group 487, the median age was 57.3 years (IR 59.7 years), whereas patients in group 488 were significantly younger, with a median age of 49.9 years (IR 42.6 years) (*p* < 0.001).

Throughout the study period, a total of 8602 admitted patients (16.3%) were under 5 years old, and 35% were older than 65 years. These two age groups comprised the majority of inpatients in each of the diagnostic groups. In group 487, the highest percentage of admissions corresponded to the age group over 65 years of age with 41.6%, whereas in group 488 the age group over 65 years of age accounted for only 29.3% of the admissions. Group 488 had a higher percentage of patients in the 15 to 44.9-year-old and in 45 to 64.9-year-old groups than group 487. The differences were significant (*p* < 0.001) and are shown in Table 1. Data related to over aging groups like all patients over 75 years and all patients over 85 years are also shown in Table 1.

**Table 1:** Hospital admissions with either clinical diagnosis or diagnosis of influenza (ICD-9487 and 488), 2009–2010 to 2014–2015 seasons

Age group Years (y.)	Number of admissions (%)			Average annual incidence × 100,000 people (95% CI)			Number of Deaths (%)			Mortality rate × 100,000 people (95% CI)			Fatality (%) (95% CI)		
	487	488	All	487	488	All	487	488	All	487	488	All	487	488	All
< 5 y.	4543 18.5%	4059 14.3%	8602 16.3%	31.8 (30.8–32.7)	28.4 (27.5–29.2)	60.1 (58.86–61.4)	15 1.5%	27 1.7%	42 1.7%	0.1 (0.05–0.16)	0.19 (0.12–0.26)	0.29 (0.2–0.38)	0.3 (0.2–0.5)	0.7 (0.4–0.9)	0.5 (0.34–0.64)
5–14 y.	1461 6%	1525 5.4%	2986 5.6%	5.3 (5–5.5)	5.5 (5.2–5.8)	10.7 (10.34–11.11)	16 1.6%	15 1%	31 1.2%	0.06 (0.03–0.09)	0.05 (0.03–0.08)	0.11 (0.07–0.15)	1.1 (0.7–1.6)	1.0 (0.5–1.5)	1.0 (0.67–1.4)
15–44 y.	3658 14.9%	6985 24.6%	10,643 20.1%	3.1 (3–3.2)	6.0 (5.8–6.1)	9.1 (8.92–9.26)	58 5.9%	230 14.8%	288 11.3%	0.05 (0.04–0.06)	0.2 (0.17–0.22)	0.25 (0.22–0.27)	1.6 (1.2–2)	3.3 (2.9–3.7)	2.7 (2.4–3.01)
45–64 y.	4652 19%	7479 26.4%	12,131 22.9%	6.4 (6.2–6.5)	10.2 (10–10.5)	16.6 (16.29–16.88)	176 17.8%	480 30.9%	656 25.8%	0.24 (0.21–0.28)	0.66 (0.6–0.71)	0.9 (0.83–0.97)	3.8 (3.2–4.3)	6.4 (5.9–7)	5.4 (5.01–5.81)
Over 65 y.	10,213 41.6%	8309 29.3%	18,522 35%	20.6 (20.2–21)	16.8 (16.3–17.1)	37.3 (36.81–37.88)	725 73.2%	803 51.6%	1528 60%	1.46 (1.36–1.57)	1.62 (1.51–1.73)	3.08 (2.93–3.24)	7.1 (6.6–7.6)	9.7 (9–10.3)	8.6 (7.85–8.65)
Over 75 y.	6923 28.2%	5056 17.8%	11,979 22.7%	27.3 (26.6–27.9)	19.9 (19.4–20.5)	47.2 (46.33–48.02)	549 55.5%	527 33.9%	1076 42.3%	2.16 (1.98–2.34)	2.08 (1.9–2.25)	4.24 (3.98–4.49)	7.9 (7.3–8.6)	10.4 (9.6–11.3)	9.0 (8.47–9.49)
Over 85 y.	2492 10.2%	1587 5.6%	4079 7.7%	35.4 (34–36.8)	22.5 (21.4–23.7)	57.9 (56.16–59.71)	262 26.5%	193 12.4%	455 17.9%	3.72 (3.27–4.17)	2.74 (2.35–3.13)	6.46 (5.87–7.06)	10.5 (9.3–11.7)	12.2 (10.6–13.8)	11.2 (10.19–12.12)
Total	24,527 100%	28,357 100%	52,884 100%	8.7 (8.6–8.8)	10.1 (9.9–10.2)	18.8 (18.59–18.91)	990 100%	1555 100%	2545 100%	0.35 (0.33–0.37)	0.55 (0.52–0.58)	0.9 (0.87–0.94)	4.0 (3.8–4.3)	5.5 (5.2–5.8)	4.8 (4.63–4.99)

The hospitalization rate for the entire period was 18.75 cases per 100,000 people (95% CI 18.59–18.91). Likewise, a different admission rate was observed by age group. The group younger than 5 years of age had the highest admission rates with 60.13 per 100,000 people (95% CI 58.9–61.4), and the group from 15 to 44.9 years of age had the lowest admission rates with 9.09 per 100,000 people (95% CI 8.9–9.3). Table 1 shows the hospitalization rates corresponding to each diagnostic group by patient age.

**Fig. 1:**
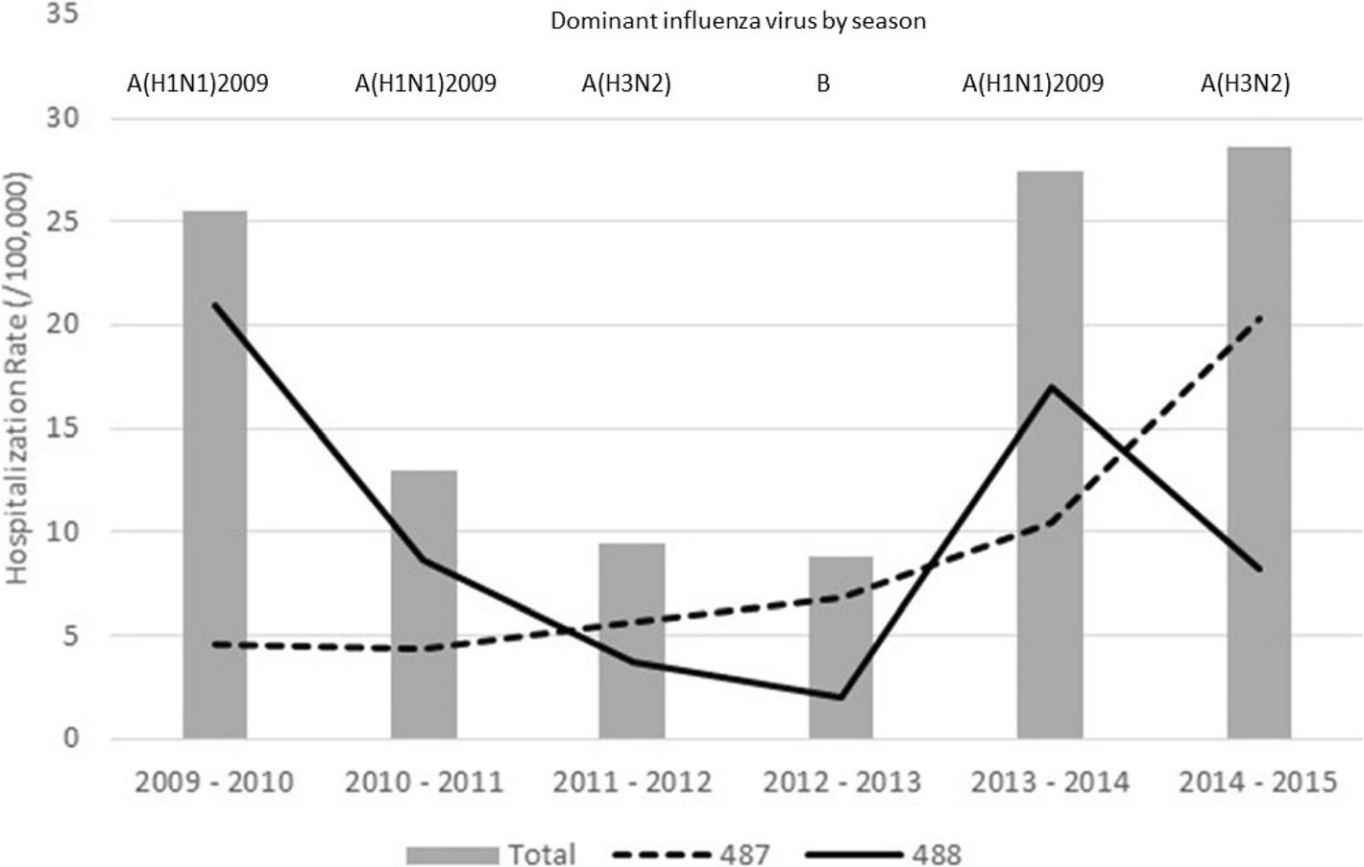
Hospitalization rate between both diagnostic groups (ICD9 487 and ICD9 488) 2009–2015

Different hospitalization rates were also observed throughout the different seasons. The lowest hospitalization rate was observed in the 2012–2013 season with 8.82 (95% CI 8.55–9.02), whereas the highest rate of 28.58, (95% CI 28.10–29.07) was observed in the 2014–2015 season. Figure 1 shows the differences in the hospitalization rates between both diagnostic groups throughout the 6 years of the study. The hospitalization rates for group 488 were higher in the 2009–2010, 2010–2011 and 2013–2014 seasons.

**Fig. 2:**
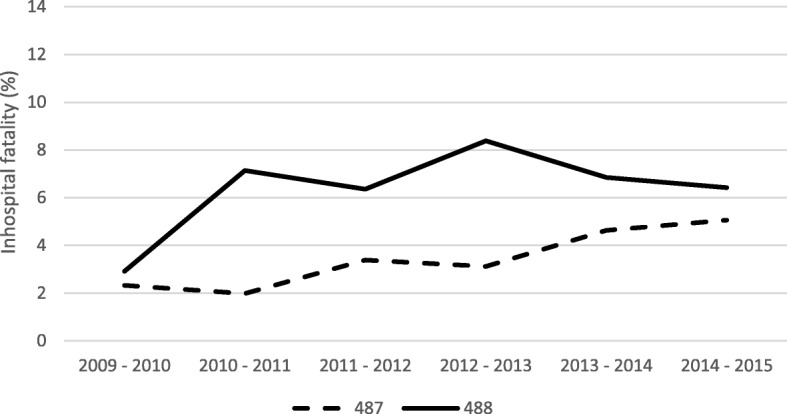
Inpatient hospital fatality (%) by season, 2009–2015

**Fig. 3:**
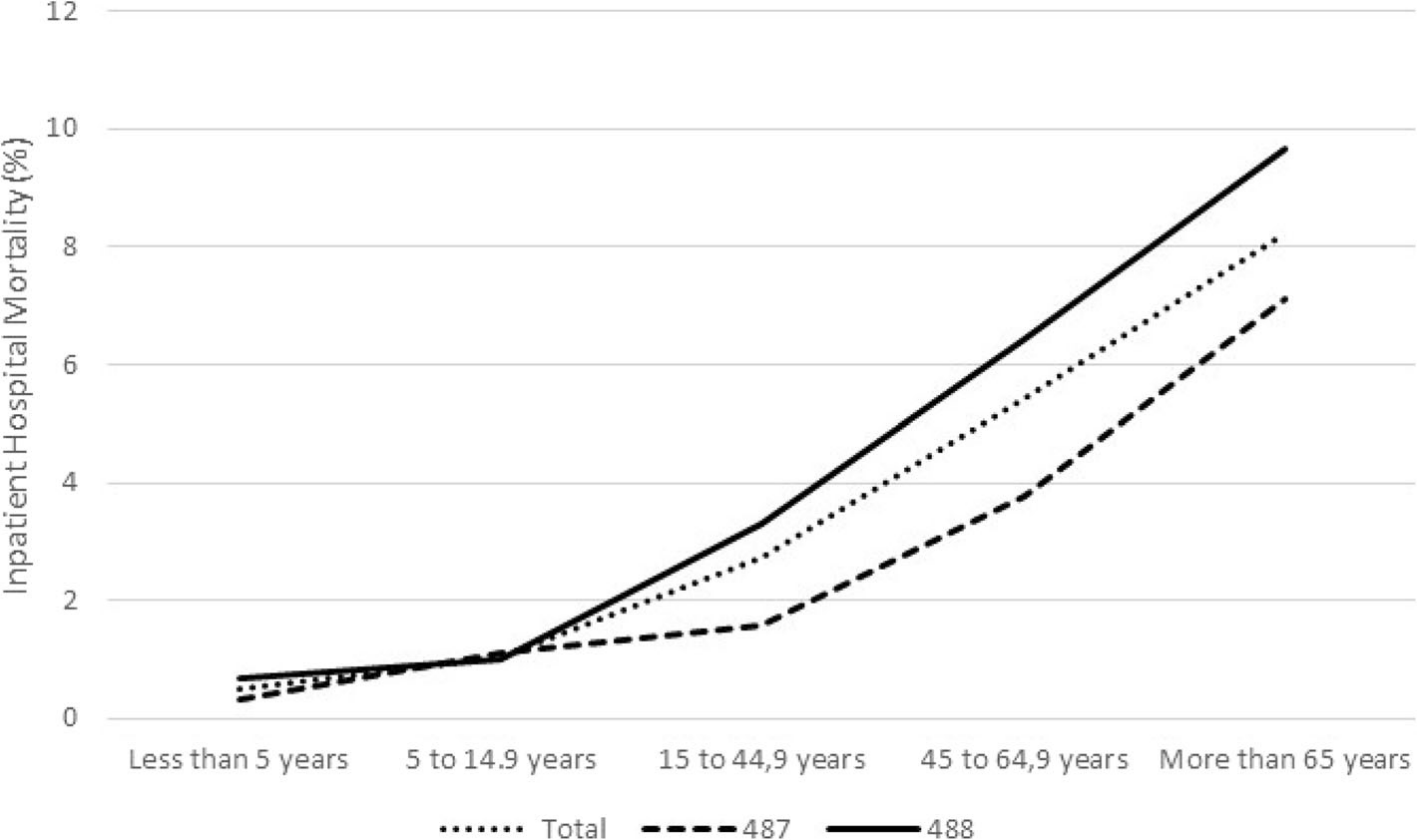
Inpatient hospital fatality (%) by age group, 2009–2015

A total of 2545 deaths were recorded among the inpatients throughout the entire period, corresponding to 5.2% of the admitted males and 4.4% of the admitted females (*p* < 0.001). The global mortality rate for the entire period was 0.90 deaths per 100,000 people (95% CI 0.87–0.94) and was highest in the group over 65 years of age with 3.08 deaths per 100,000 people (95% CI 2.93–3.24) and lowest in the group from 5 to 14.9 years of age with 0.11 deaths per 100,000 people (95% CI 0.07–0.15). Among the patients admitted, the most represented age group was over 65 years old, which reached 60% of deaths. Likewise, the group with the highest Inpatient hospital fatality was also the group over 65 years of age with 8.25 (95% CI 8.47–8.65), followed by the group 45–64.9 years of age with an inpatient hospital fatality rate of 5.41 (95% CI 5.01–5.81) and the group under 5 years of age with an inpatient hospital fatality rate of 4.81 (95% CI 4.63–4.99). Number of deaths and fatality rates relative to the age groups observed in code 487 and code 488 groups are also shown in Table 1. Group 488 presented greater inpatient hospital mortality than group 487 during all seasons and in almost all age groups except for the age group corresponding to 5–14.9 years, which had similar values (Figs. 2, 3).

In addition, other clinical conditions codified during admission were analyzed and classified. The most frequent clinical condition was “Lung disease” codified in 29,797 times (56.3%) followed by “Heart Disease” in 25.5% and “Diabetes and other endocrine disease” in a 15.8%. Inpatient hospital fatality was higher in “Renal disease” (15.2%) followed by “Dementia, Stroke” in 11.9%. Frequency of each clinical condition and Inpatient hospital fatality by diagnostic group are describe in Table 2.

**Table 2:** Frequency of each clinical condition and Inpatient hospital fatality by diagnostic group

	Number of Diagnostics (% diagnostics)			Number of Deaths (% inpatient)		
Underlying Clinical Condition	Total	ICD 487	ICD 488	Total	ICD 487	ICD 488
Enlarge spleen, anemia	7910 (15%)	3853 (15.7%)	4057 (14.3%)	693 (8.8%)	236 (6.1%)	457 (11.3%)
Cirrhosis	1487 (2.8%)	676 (2.8%)	811 (2.9%)	135 (9.1%)	50 (7.4%)	85 (10.5%)
Diabetes and endocrine disease	8335 (15.8%)	4112 (16.8%)	4223 (14.9%)	496 (6%)	213 (5.2%)	283 (6.7%)
Heart Disease	13,483 (25.5%)	6900 (28.1%)	6583 (23.2%)	1351 (10%)	569 (8.2%)	782 (11.9%)
Hematologic Cancer	2390 (4.5%)	1132 (4.6%)	1258 (4.4%)	273 (11.4%)	84 (7.4%)	189 (15%)
Immunodeficiency and organ transplant	3504 (6.6%)	1701 (6.9%)	1803 (6.4%)	201 (5.7%)	70 (4.1%)	131 (7.3%)
Lung disease	29,797 (56.3%)	11,809 (48.1%)	17,988 (63.4%)	2156 (7.2%)	795 (6.7%)	1361 (7.6%)
Non hematologic cancer	1992 (3.8%)	988 (4%)	1004 (3.5%)	209 (10.5%)	90 (9.1%)	119 (11.9%)
Nutritional deficiencies	1471 (2.8%)	757 (3.1%)	714 (2.5%)	119 (8.1%)	59 (7.8%)	60 (8.4%)
Renal Disease	7215 (13.6%)	3544 (14.4%)	3671 (12.9%)	1095 (15.2%)	398 (11.2%)	697 (19%)
Dementia, Stroke	3908 (7.4%)	2091 (8.5%)	1817 (6.4%)	464 (11.9%)	229 (11%)	235 (12.9%)
Rheumatologic Diseases	925 (1.7%)	440 (1.8%)	485 (1.7%)	53 (5.7%)	24 (5.5%)	29 (6%)

A multivariate analysis was performed attending to gender and coding group. Patients were categorized in four groups based on age and underlying conditions; a) 6987 (13.2%) patients between 2 years and 65 years without underlying clinical conditions as reference group, b) 21,661 (39.8%) patients between 2 years and 65 years with underlying clinical condition, c) 5714 patients (10.8%) less than 2 years and d) 18,522 patients (35%) over 65 years. The adjusted OR for in hospital dying were 1.48 (1.36–1.61; *p* < 0.001) for ICD 488, 1.71 (1.08–1.27; p < 0.001) for male gender, 2.17 (1.13–4.17; p < 0.001) for patients less than 2 years, 21.92 (12.93–37.19; p < 0.001) for patients between 2 years and 65 years with underlying clinical condition and 45.15 (26.66–76.49; p < 0.001) for patients over 65 years.

**Fig. 4:**
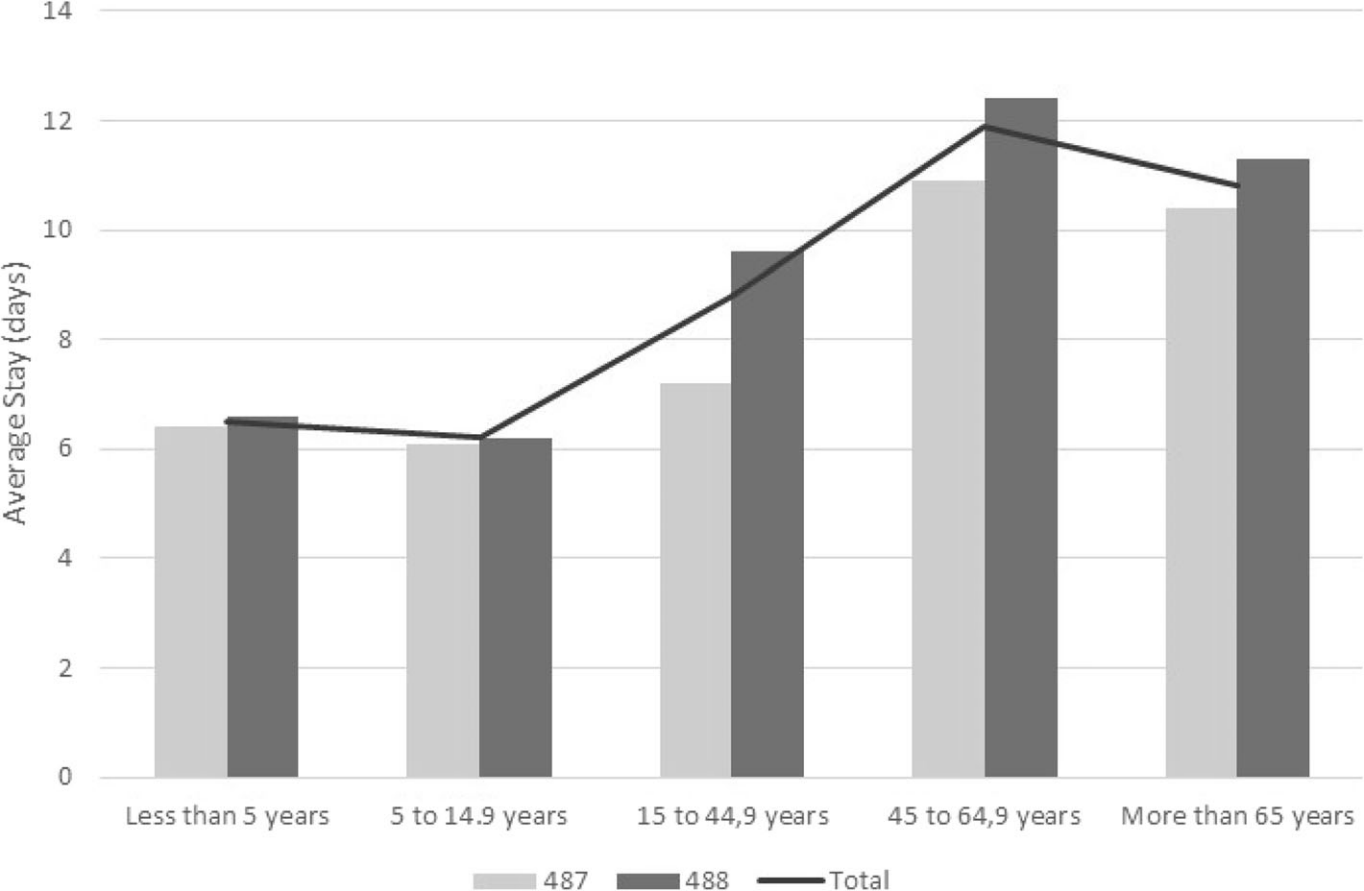
Average hospital stay (days) by age group, 2009–2015

The average inpatient length of stay was 9.68 days (95% CI 9.58–9.79), with a minimum value of 6.17 (95% CI 5.82–6.49) in the 5- to 14.9-year-old age group and a maximum of 11.86 days of stay (95% CI 11.55–12.15) in the 45 to 64.9 year old age group. The average stay was 9.02 days (95% CI 8.85–9.18) in group 487 and 10.25 days (95% CI 10.08–10.41) in group 488. The differences in the average length of stay between groups 487 and 488 by age are shown in Fig. 4 and were significant for the three groups older than 15 years of age (*p* = 0.001).

## Discussion

The burden of disease due to influenza in Spain and its neighboring countries remains very high and has a clear impact on the health system, with an overload of health services during epidemic periods and a clear increase in health spending. Although most influenza cases do not require hospital admission, influenza cannot be simply considered as a mild illness. Today, we know that influenza can be a serious and even deadly disease, especially in the most vulnerable populations (i.e., children and the elderly) and in at- risk populations.

Some authors have found a low sensitivity with ICD9-CM codes for the identification of influenza cases [7]. The sensitivity and predictive values of hospital influenza diagnostic records have been related to the prevalence of seasonal influenza and therefore the sensitivity of the clinician proceeding with the diagnosis and there might not be useful for the identification of early epidemic outbreaks or epidemiological surveillance [8]. However, these records have been recently suggested to provide information for population surveillance by improving the specificity according to the case definition and the codes selected [9]. If co-circulation or other respiratory viruses are present the hospitalization rate may be overestimate. In the other hand, an underestimation of the hospitalization is also possible due to lack the specificity and sensitivity of the rapid test. It has been estimated that the sensitivity of influenza-specific ICD-9 code 487 was 65% (95 CI; 61–68%) and the positive predictive value was 88% (95 CI; 84–90%) [10]. Ohers authors have found a good correlation between some ICD-10 codes and the diagnosis of influenza in small samples and during peaks of epidemic activity, such as occurred in 2009 [11]. In fact, ICD10 has the J9 - J11 coding for influenza due to certain identified influenza viruses (J9), due to other identified influenza virus (J10) and due to unidentified influenza virus (J11) thereby contributing to a better specificity and sensitivity but this coding has not been available in Spain until 2016.

In our study, we found significantly high hospitalization rates. The Spanish National Centre for Epidemiology (SNCE), report an accumulated hospitalization rate of severe hospitalized confirmed influenza cases (SHCIC) adjusted for age of 5.97 (5.68–6.26) cases per 100,000 population in the season 2010–2011 and 11.53 (11.09–11.99) in season 2013–2014 and a minimum of 2.58 (2.36–2.80) cases per 100,000 population in season 2012–2013 [12]. Data are not comparable because SNCE defines SHCIC all cases of patients with severe clinical symptoms as a laboratory-confirmed diagnosis of influenza report [13]. However, since season 2017–2018 the SNCE begun the surveillance of confirmed hospitalized cases of influenza, regardless of their severity, to evaluate the real impact that influenza have in the hospitalization of cases [14].

The distribution of inpatients in the two groups was different throughout the study. As shown in Fig. 1, during the 2009–2010, 2010–2011 and 2013–2014 seasons, an increase in coding for group 488 was observed, corresponding to the virus that was predominantly circulating in our territory [A(H1N1)2009], whereas in 2011–2012, 2012–2013 and especially 2014–2015, greater codification was observed for group 487. The circulating virus was A (H3N2) in the 2011–2012 and 2014–2015 seasons and the B virus in the 2012–2013 season [15]. However, severity of strains can vary from year to year and there could be over codification depending of previous seasons in detriment of sensitivity and specificity.

Recent data suggests that seasonal influenza-associated respiratory deaths has been underestimated and has increased from 290,000 to 645,000 cases worldwide [16]. In our study, the difference in inpatient hospital fatality between the coding groups increased significantly with the age of the patients. Similarly, to the hospitalization rates, estimating the real value of the fatality associated with inpatients is difficult. Total deaths associated with influenza have been estimated to be up to four times higher if cardiovascular and respiratory complications are considered because direct deaths from pneumonia in which influenza is not included in the diagnosis may also be responsible.

In a meta-analysis, Wong et al. reviewed the risk of fatality of influenza virus A (H1N1 pdm09) [17] when the virus was defined as the risk of death between cases. However, the methodological differences found in the literature, especially in the case definition, complicate the comparison between symptomatic cases and those confirmed by a laboratory. In a subsequent meta-analysis [18], the authors analyzed the hospitalization fatality risk (HFR) based on the number of deaths among patients admitted with influenza that was confirmed by a laboratory. Among other estimates, they observed a constant increase in hospital fatality with age, from ≤6% in children to 6–30% in the elderly. In our study, the group younger than 5 years of age had a significantly lower fatality rate (0.33 for group 487 and 0.67 for group 488) than those (7.1 and 9.7, respectively) observed for the group over 65 years of age. Likewise, in our country, the surveillance system for severe hospitalized confirmed cases of influenza for the seasons following the 2009 pandemic, which also refers to patients with a severe clinical picture with a laboratory diagnosis, found a higher inpatient hospital fatality rate associated with an increase in age [15]. This behavior has been described in previous studies [19, 20] and indicates a role of influenza as a probable cause for the development of complications in patients with high comorbidity, as has been observed in older adults. In fact, in our study, underlying clinical diagnostics, described as risk groups for vaccination, increase in hospital mortality rates.

## Conclusions

In conclusion, in our study, influenza diagnosis was present in a significant number of hospital admissions. The code used for diagnosis (ICD-9-CM 488), male sex, age groups and associated risk clinical conditions showed a direct relationship with inpatient hospital fatality. For this reason, prevention strategies through vaccination with a wide coverage of groups/serotypes are an essential component to solve the problem together with epidemiological surveillance of the disease, which is the key strategy for the control of epidemics and pandemics.

## Data Availability

The datasets analyzed in this study belong to the Spanish Ministry of Health, Consumer Affairs and Social Welfare and can be found in https://www.mscbs.gob.es/estadEstudios/estadisticas/cmbd.htm. No granted permission is needed to access the raw data. Researchers can access the database by filling the necessary form (http://www.mscbs.gob.es/estadEstudios/estadisticas/estadisticas/estMinisterio/SolicitudCMBD.htm).

## Abbreviations

CMBD: Mínimum Basic Data Set [Conjunto Mínimo Básico de Datos]
ICD-XX CM: International Classification of Diseases, Clinical Modification
SHCIC: Severe Hospitalized Confirmed Influenza Case
SNCE: Spanish National Centre for Epidemiology
